# The Role of Native-Language Knowledge in the Perception of Casual Speech in a Second Language

**DOI:** 10.3389/fpsyg.2012.00249

**Published:** 2012-07-13

**Authors:** Holger Mitterer, Annelie Tuinman

**Affiliations:** ^1^Max Planck Institute for PsycholinguisticsNijmegen, Netherlands

**Keywords:** /t/ reduction, casual speech processes, L2, speech perception, lexical and syntactic constraints

## Abstract

Casual speech processes, such as /t/-reduction, make word recognition harder. Additionally, word recognition is also harder in a second language (L2). Combining these challenges, we investigated whether L2 learners have recourse to knowledge from their native language (L1) when dealing with casual speech processes in their L2. In three experiments, production and perception of /t/-reduction was investigated. An initial production experiment showed that /t/-reduction occurred in both languages and patterned similarly in proper nouns but differed when /t/ was a verbal inflection. Two perception experiments compared the performance of German learners of Dutch with that of native speakers for nouns and verbs. Mirroring the production patterns, German learners’ performance strongly resembled that of native Dutch listeners when the reduced /t/ was part of a word stem, but deviated where /t/ was a verbal inflection. These results suggest that a casual speech process in a second language is problematic for learners when the process is not known from the leaner’s native language, similar to what has been observed for phoneme contrasts.

## The Role of Native-Language Knowledge in the Perception of Casual Speech in a Second Language

Speech perception studies are often performed under ideal circumstances, with participants listening to their native language in a sound-proof booth. Experimental stimuli have usually been recorded under similarly optimal circumstances by a speaker, who is carefully reading out loud. Outside the laboratory, the situation is, however, often less ideal. Environmental noises are common, speakers are less careful than during reading, and the language we listen to might not be our native language. All of these influences make speech perception harder.

To start, speech perception in a second language is notoriously difficult. Non-native listeners have the disadvantage that the second language (L2) often contains phoneme contrasts that they are unfamiliar with given their native language (L1). A large body of research (see, e.g., Strange, 1995; Bohn and Munro, 2007 for an overview). has shown that mismatches between the phoneme repertoires of the L1 and L2 cause perceptual difficulties. A classical example is the segmental contrast between /r/ and /l/ in English, which difficult for Japanese listeners because these speech sounds match a single Japanese category equally well, leading to perceptual difficulties (e.g., Underbakke et al., 1988; Bradlow et al., 1997; Ingram and Park, 1998; Cutler et al., 2006). Cutler and Otake (2004), tested auditory repetition priming with English /r/-/l/ minimal pairs – such as *right-light –* and Japanese listeners. This contrast is difficult for these listeners and consequently priming was observed.

These difficulties of L2 listeners are not limited to minimal pairs. Research in psycholinguistics has shown that the recognition of any word entails activation of multiple word candidates which compete for recognition: For example, when listeners hear the word *captain*, not only the word *captain* is activated, but also words such as *cap* and *capitol* are temporarily activated (Davis et al., 2002). During this process, difficult L2 contrasts create additional competitors for non-native listeners. For instance, Dutch learners of English, who have a hard time to distinguish the vowels /ε/ and /æ/, activate the word *deaf* /dεf/ when hearing the longer word *daffodil* /dæf?dil/ (Broersma and Cutler, 2011).

As this shows, listening to a non-native language is, compared to L1 listening, an adverse condition by itself. Non-native listeners are confronted with difficult segmental contrasts leading to spurious lexical activation. To make matters worse, L2 perception seems to be more strongly affected by additional challenges. For example, several studies have shown that, in the presence of background noise, speech perception by non-native listeners is more strongly affected than that of native listeners (see Lecumberri et al., 2010, for an overview). Non-native listeners do not only require a better signal-to-noise ratio (SNR) for the recognition of speech in noise, they also need larger changes in the SNR to recover (i.e., their psychometric functions of recognition rate over noise levels are shallower than those of native listeners, see Van Wijngaarden et al., 2002).

However, as indicated above, noise-masking is not the only challenge for speech perception. Spontaneous speech is also much harder to comprehend than carefully read speech. Little research, however, has focused on how non-native listeners deal with the adverse conditions created by casual speech. Is casual speech, like background noise, especially difficult to overcome for non-native listeners?

This is an important question. After all, casual speech is what, as language users, we mostly hear and produce. This speech is extremely variable as is evident from casual speech corpora from American English (Dilley and Pitt, 2007; Pitt et al., 2007), Dutch (Oostdijk, 2000; Pluymaekers et al., 2005), and French (Torreira et al., 2010). In these corpora, processes such as assimilation, epenthesis and extreme reduction occur frequently and result in pronunciation variations and potential ambiguities. For instance, consonant reduction such as the deletion of /t/ in *lost* confronts listeners with unintended words, in this case *loss*. The question how listeners deal with the resulting non-canonical forms in perception has recently received widespread attention. Alterations can often (temporarily) mislead listeners, and result in word recognition being harder than it would have been for the canonically pronounced versions (Ernestus et al., 2002; Sumner and Samuel, 2005, 2009; Tucker and Warner, 2007; Warner et al., 2009). But native listeners are still able to cope with the extreme variability that occurs in spontaneous speech, for a large part because they make of use information conveyed by phonetic detail and context. With regard to phonetic detail in compensation for place assimilation, listeners are able to distinguish the /p/ of English *ripe* in *ripe berries* from the assimilated final /t/ of *right berries* (Gow, 2002), even though the classical phonological analysis (c.f. Gussenhoven and Jacobs, 1998) prescribed both to be transcribed as [p] (see Spinelli et al., 2003, for a similar example). With regard to context, listeners make use of the fact that most reductions are conditioned by phonological context (Gaskell and Marslen-Wilson, 1996; Gow, 2003; Mitterer and Blomert, 2003; Mitterer and McQueen, 2009), and are more likely to infer an altered or deleted segment in contexts that facilitate reduction.

However, all of this research has been carried out with native listeners. Given that even these experienced listeners are often burdened by reductions, what is going to happen when non-native listeners hear the same sort of input? Hear it they will, because L2 listeners cannot permanently confine themselves to speech situations in which the input is as close to canonical perfection as it is in the classroom or on language tapes. Therefore, we investigate here how casual speech processes affects L2 listening.

Obviously, not all non-native casual speech processes may be equally difficult for a non-native listener, just as not all non-native phoneme contrasts are equally difficult. With regard to phoneme contrasts, for instance, the Perceptual Assimilation Model (Best and Tyler, 2007) assumes different types of contrast relations, which are more or less difficult for the learner. In short, contrasts that are similar in L1 and L2 pose little problems for L2 learners. If this also holds for casual speech processes, casual speech processes that occur in the L2 but not in the L1 should be particularly hard for non-native listeners. In line with this assumption, Tuinman et al. (2011) show that Dutch listeners have trouble with the process of /r/-intrusion in their L2 English, in which an /r/ is inserted between two vowels at a word boundary [as in “I saw(r) a film today” in the Beatles’ song *A day in the life*]. As this /r/-insertion does not occur in Dutch, it should hence be difficult for Dutch learners, and, indeed, it is. However, it is unclear if the reverse is also true. Does a L2 casual speech process cause less of a problem if it does occur in the L1?

To answer this question, we tested whether German advanced learners of Dutch are able to compensate for /t/-reduction in Dutch. There are two reasons to choose the process of /t/-reduction. The first is that the process of /t/-reduction is found in many languages (Guy, 1980) and patterns very similarly in the Germanic languages English, German, and Dutch, so that German learners are familiar with /t/-reduction from their native language experience. Second, /t/-reduction in Dutch has been intensively studied (Mitterer and Ernestus, 2006; Janse et al., 2007; Mitterer and McQueen, 2009). Based on these studies, we focus on three aspects that have been shown to influence compensation for /t/-reduction by Dutch L1 listeners: phonetic detail, preceding phonological context, and higher-level knowledge, such as lexical and syntactic knowledge. First of all, Dutch allows various gradations of /t/-reduction, and listeners are highly sensitive to the subtle differences in phonetic detail that result from this. Secondly, preceding context is important as /t/-reduction is more likely after /s/ than after /n/ in Dutch and listeners take this into account in perception. Finally, listeners also make use of higher-level knowledge, and are more likely to restore a reduced /t/ if this “leads to a word.” That is, they are more likely to report a /t/ at the end of “fros…” (*frost* being a word) than at the end of “blis…” (*blist* being an English non-word).

However, before we can use /t/-reduction as a case study we need to know exactly how similarly it patterns in Dutch and German. Here, it is important to note that /t/-reduction is more likely the more informal the speech. Mitterer and Ernestus (2006) found a much higher incidence of /t/-reduction in face-to-face conversations than in audio book recordings. This means that the pre-existing spontaneous speech corpora for Dutch and German cannot be used for a quantitative comparison, because it is difficult to judge whether the corpora are similar in formality (e.g., the German Kiel Corpus is based on a business-appointments scenario, and interlocutors address each other with the honorific “*sie*” form). Therefore, we decided to run a production study, which allows us to exert the level of control necessary for a quantitative comparison.

Critically, we will focus on whether /t/-reduction in German is more likely after /s/ than after /n/, which is the pattern attested for Dutch (Mitterer and Ernestus, 2006). Mitterer et al. (2008) had shown that, in perception, listeners take into account the context, that is, they are more likely to perceptually restore a reduced /t/ after /s/ than after /n/. This pattern is partially perceptually motivated: Listeners in fact have a hard time to hear the reduction of /t/ after /s/ (see Steriade, 2001; Mitterer et al., 2006a, for elaboration of such perceptual motivations of phonetic reductions). However, language learning also seems to play a role for this context effect, making it an interesting candidate for L2 effects.

Additionally, we investigated the pattern of /t/-reduction in nouns – in which the /t/ was part of the word’s stem – and /t/-reduction in verbs, in which /t/ is a verbal inflection for the third-person singular. There is evidence to suggest that morphological variables influence reduction processes (Guy and Boyd, 1990). The morphological influence may be different for German than for Dutch, because German is morphologically richer with many different verb conjugations, a three- rather than a two-gender system, and more grammatical cases for nouns. This is also the case for verb inflection. Where German uses four different conjugations (first sing: -e, second sing: -st, third sing, and second pl: -t, first and third pl: -en), Dutch has only three conjugations which are also more consistently used, with a single conjugation for all plurals (-en) and a simpler scheme for the singular (first: Ø, second and third: -t).

## Experiment 1

In order to elicit casual speech in a controlled setting we used a blending task for nouns and a conjugation task for verbs. Both have previously been used successfully to elicit connected speech (Stephenson and Harrington, 2002; Zimmerer and Reetz, 2011). The tasks ask participants to produce a sentence that is only cued by words on the screen, that is, it is not a reading task. Instead, the tasks were tailored in such a way that participants had to formulate a sentence by themselves in order to draw attention away from the enunciation.

### Method

#### Participants

Ten Dutch and 10 German speakers took part in this experiment. The Dutch participants were students at the University of Nijmegen, the Netherlands and members of the Max Planck Institute’s subject pool. Some of the German participants were also taken from this population; others were employes at the Max Planck Institute with basic knowledge of Dutch. The German participants had on average 2.7 years of exposure to Dutch and more than 10 years of exposure to English. Participants were asked in what proportions they used their languages for study/work, in peer-groups and with family. Dutch was the main language used for study/work purposes (74%), while language use with peer-groups was slightly dominated by German (49 vs. 41% for Dutch). The Dutch participants all had some formal teaching in German (5 years), but indicated to not use German in either study, peer-groups, or family, with the exception of one participant who used German occasionally (10%) for study purposes. None of the participants reported any hearing loss. All were volunteers and received a small fee for participation.

#### Materials, design, and procedure

The experiment consisted of two production tasks and was constructed in such a way that both groups of participants performed the same tasks. The experiment was run on a standard PC running with the NESU package to control stimulus presentation. Participants were tested one at a time in a sound-proof booth. They sat at a comfortable reading distance from the computer screen and had a microphone and a two-button response box in front of them.

The first part of the experiment examined /t/-reduction in verbs with a sentence generation task. Participants saw words in random order on the computer screen (e.g., in Dutch, *bij/Maarten/de bushalte/wonen*, Engl., “close to/Maarten/the bus stop/live”). They were instructed to produce a sentence with the third-person singular present – which ends on /t/ in both Dutch and German – using these words. The stem of the critical verb ended on either /n/ or /s/ and the verb was always presented in its infinitive form. In the example, the correct response was *Maarten woont bij de bushalte* (Engl., “Maarten lives close to the bus stop”). The words following the verb always started with a consonant to prevent resyllabification of the verb-final /t/ to the onset of the following word. Participants received 40 different stimulus sentences. The presentation of the sentences was random and different for every participant.

The second part of the experiment tested /t/-reduction after /n/ and after /s/ in proper names, using a blending task. Participants saw two non-existent place names (e.g., *Toestwoud* and *Liekbeek* for Dutch), and made a new place name with the first part of the first place name and the second part of the second place name (in this case *Toestbeek*). The first part of the place name ended in either /nt/ or /st/, so that half of the /t/s were preceded by /n/ and the other half by /s/. The new place name had to be produced in a sentence frame cued by the name of a store (e.g., *groenteboer/Gemüsehändler*, “greengrocer”) and a product (e.g., *appels/Äpfel*, Engl. “apples”), and the intended sentence was *Bij de groenteboer in Toestbeek koop ik appels* (“At the greengrocer in *Toestbeek* I buy apples”). Again, participants received 40 different sentence frames and the presentation of the sentences was random and different for every participant.

Both parts started with four practice trials. Each trial (experimental and practice trials) began with a blank screen. Then, the words were presented on the screen. After 1500 ms the message “Press the right button to continue” was displayed on the screen in either Dutch or German, so that participants could continue with the next sentence.

### Results

The 1600 sentences (10 speakers of each language × 80 tokens) were analyzed for /t/-reduction in the critical words. On the basis of visual inspection of the sound files using PRAAT (Boersma and Weenink, 2005), the productions of /t/ were classified and it was judged whether the /t/ was present or not. The classification was done in accordance with the method employed in Mitterer and Ernestus (2006), who distinguished five variants of /t/ (canonical, weak and strong frication, closure-only, and complete deletion, see Figure 2 for re-synthesized examples). After this initial classification, the first two signals (full /t/ and strong frication) were then coded as present, the other as deleted. One may wonder why the coding scheme does not include the option of glottal stops as a phonetic correlate of an underlying /t/ (the glottal stops can sometimes replace /t/ in German). An analysis of the German Kiel Corpus (IPDS, 1994) shows, however, that the glottal stop is almost exclusively used for /t/ in a pre-schwa position (accounting for more than 93% of all /t/-glottal stop replacements), so it is unlikely to be frequent in the current environment.

**Figure 1:**
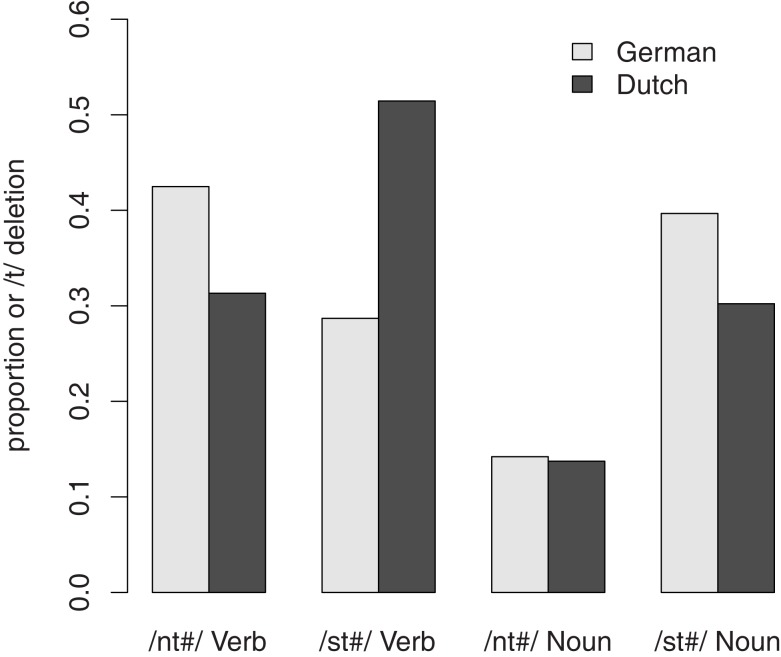
**Proportion of /t/-deletion in the same production tasks (Experiment 1) by Dutch and German speakers**.

Figure 1 shows the results of the transcription. Overall, /t/ was deleted in 25% of the nouns and 40% of the verbs. This indicates that the tasks were successful in focusing attention away from careful enunciation, allowing the application of optional casual speech processes. It is also clear that rate of /t/-reduction was not the same for all conditions. Dutch participants tend to delete /t/ more often if the /t/ was preceded by an /s/ rather than an /n/. This pattern was consistently observed for both nouns and verbs. German participants, however, show a different pattern for verbs, with /t/-reduction in fact being more likely in verbs with an /nt/-coda than in verbs with a /st/-coda.

For the statistical analysis, the results were analyzed separately with a linear mixed-effects model with a binomial linking function to account for the categorical nature of the dependent variable (c.f. Dixon, 2008). Participant and item were entered as random factors with a maximal random effect structure and Noun/Verb, Native Language and Preceding Context as fixed factors. The fixed factors were contrast-coded, with the preceding context /n/ and the Native Language German coded as −0.5. A positive regression weight hence would indicate that more /t/s were produced in the /s/-context, and by the Dutch participants. In contrast coding for binary variables, one level is coded as −0.5 and the other as 0.5, so that regression weights for simple effects in the regression models show the overall effect of a given variable for the complete data set (c.f. Barr, 2008). This coding has the advantage that the predictors for the main effects are linearly independent from the predictor for the interaction. The analysis with all three factors showed a significant three-way interaction (*b* = 2.7, *z* = 1.97, *p* < 0.05). To understand the nature of this interaction, separate analysis were performed for nouns and verbs.

Table 1 shows the regression weights for the regression models for the proper names and verbs. For proper names there were no significant differences between Dutch and German speakers. The Preceding Context did have a significant effect in that /t/ was more often reduced after /s/ than after /n/. For verbs, there were no main effects of Native Language or Preceding Context, but a significant interaction. Separate analyses for German and Dutch speakers showed that only the Dutch speakers have a significant effect of Preceding Context and reduced /t/ more often after /s/ than after /n/ (b = −1.89, *p* < 0.05), while no such effect was observed for German speakers (*p* > 0.1).

**Table 1:** **Experiment 1: Regression weights for the models for nouns and verbs**.

Type of word	Effect	Regression weight (SE)
Proper name	(Intercept)	2.79 (0.42)***
	Native language	−0.22 (0.81)
	Preceding context	−2.48 (0.67)***
	Native language: preceding context	−0.39 (1.23)
Verb	(Intercept)	1.00 (0.40)*
	Native language	0.38 (0.79)
	Preceding context	−0.62 (0.57)
	Native language: preceding context	−2.34 (1.12)*

***p < 0.001; **p < 0.01; *p < 0.05. Significance levels are based on the *z*-statistic. The predictors are contrast-coded (Native Language: Dutch = −0.5, German = 0.5, Preceding Context: /n/ = −0.5, /s/ = 0.5).

### Discussion

The results show that Dutch and German are similar but not identical with regard to /t/-reduction. In both languages, /t/-reduction was more likely after /s/ than after /n/. This replicates the results in the corpus studies of Mitterer and Ernestus (2006), and therefore raises the question why /t/ is likely to be reduced after /s/. One possibility is that /t/ is more predictable after /s/ than after /n/, making it more prone to deletion according to hypothesis such as the “Smooth Signal Redundancy Hypothesis” (Aylett and Turk, 2006). To evaluate that, we calculated the type and token frequency in the of the codas /n/ vs. /nt/ and /s/ vs. /st/ using frequencies from the SubtLex Corpus (Keuleers et al., 2010). As it turns out the ratios (Frequency of /Ct/ divided by the Frequency of /C/) indicate that the likelihood of a coda with /t/ is higher for /n/ than for /s/ (Types: 1.54 vs. 0.16; Tokens: 0.14 vs. 0.10). That is, a /t/ is more predictable after /n/ than after /s/. Mitterer et al. (2008) have provided another explanation for the tendency to reduce /t/ especially after /s/. They argued that this is an example of a perceptual constraint that imposes itself on production. The reduction of /t/ is less salient after the spectrally similar /s/ than after the spectrally dissimilar /n/.

Our main focus was, however, to investigate the role of morphological differences. In line with our suspicion, reduction of a morphological /t/ was different in German than in Dutch. In Dutch, verbs patterned just as nouns, with more reduction after /s/ than after /n/ but, in German, /t/-reduction was independent of the preceding context. A possible explanation for this pattern is that the morphological /t/ has no special status in Dutch, a morphologically less rich language than German, and hence does not differ from a /t/ that has no morphological role. In the morphologically richer language German, however, the morphological status seems to block the influence of the preceding phonological context.

What do these results mean for the perception of /t/-reduction in Dutch by German learners? If the similarity of the L1 and the L2 pattern governs the perception of casual speech processes in L2, German listeners may be able to perform very similarly to native Dutch listeners for cases in which the /t/ has no morphological role. Then, the L1 knowledge of German learners matches the Dutch L2. However, /t/-reduction in verbs may be more of a challenge for German learners of Dutch, because, here, the L1 knowledge does not fit the pattern of the L2 that well.

To investigate this, we presented German and Dutch listeners with varying amounts of acoustic evidence for word-final /t/ in verbs, nouns, and adjectives. Five realizations of /t/, from full production to complete deletion, are presented in two acoustic contexts, after /n/ (where /t/-reduction is unlikely) and after /s/ (where /t/-reduction occurs frequently). These five levels of the /t/-Ø continuum are based on findings from a corpus study on word-final /t/ in Dutch (Mitterer and Ernestus, 2006). In each sentence, listeners judged whether the target word ended in /t/ or not.

If non-native listeners have difficulty in exploiting the phonological cues for /t/, they might base their judgments of whether the target word ended in a /t/ or not on higher-level information such lexical status and syntax in verbs. Therefore, we also added as a factor whether the /t/ is “prescribed” by syntax or lexical status. In Experiment 2, the /t/ had no morphological role, but was part of the stem of a noun or an adjective, and lexical information prescribed the presence of a /t/ (*charmant* “charming,” *charman* being a Dutch non-word) or not (*kanon*, “gun,” *kanont* being a Dutch non-word). In Experiment 3, target words were verbs (e.g., *ren* “run,” *kus* “kiss”) which makes it possible for listeners to use grammar to predict whether or not the ending should be /t/. The Dutch present tense third-person singular inflection is /t/ (e.g., *zij rent*, “she runs”) while the first-person inflection is null (e.g., *ik ren*, “I run”). Hence, listeners should be biased to expect a /t/ at the end of a verb that is preceded by the third-person singular pronoun *zij* and biased to expect no /t/ at the end of a verb that is preceded by the first-person singular pronoun *ik*.

## Experiment 2

In this experiment, we investigated the perception of reduced /t/. As materials we used synthesized speech (following Mitterer and Ernestus, 2006). It may be argued that this defeats our purpose to investigate speech perception in ecologically valid listening situations. However, Mitterer and McQueen (2009) have shown that all the basic effects reported by Mitterer and Ernestus (2006) with synthetic speech can be replicated with natural speech. As argued in Mitterer and McQueen, 2009, p. 258), the use of synthetic speech hence does not lead to ecologically invalid results, as long as the acoustic patterns are based on patterns observed in spontaneous speech (as done by Mitterer and Ernestus, 2006). Stated otherwise, the unusual voice characteristics of synthetic speech do not seem to influence how listeners deal with reduced speech.

### Method

#### Participants

Sixteen native speakers of German participated in the experiment. The participants were students at the University of Nijmegen, the Netherlands and members of the Max Planck Institute’s subject pool. None reported any hearing loss. All were volunteers and received a small fee for participation. The German participants had a high level of proficiency in Dutch as L2 as they followed an intensive Dutch language course and passed an exam as a requirement to enter a Dutch university. To compare the data to native performance, control data of Dutch native speakers were taken from Mitterer and Ernestus (2006).

#### Materials

**Figure 2:**
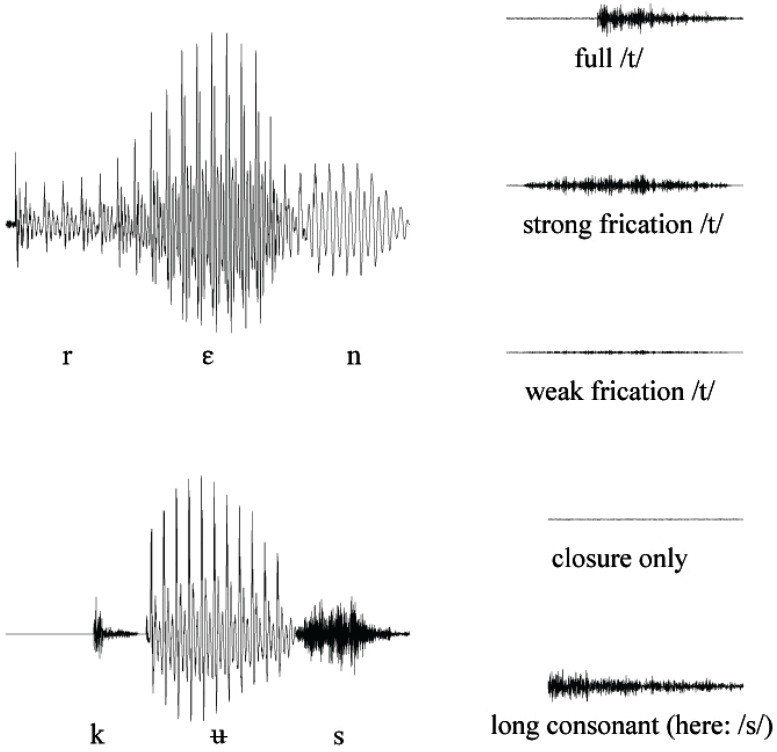
**Realizations of word-final /t/ in four Dutch casual speech utterances from full production (top row) to deletion (bottom row)**. See text for details.

The materials for the experiment were those of Mitterer and Ernestus (2006), Experiment 2. In this experiment, target words were embedded in simple sentence frames such as “Wim sprak *target* nauwelijk uit” (Eng. “Wim hardly pronounced *target”*). The name of the speaker and the adverb (here: hardly) changed over sentences. The stimulus sentences were created by synthesis via a Klatt synthesizer (Klatt, 1980). The target stimuli were the non-words *orkes* and *charman*, as well as the Dutch words *moeras* and *kanon*, followed by one of five coda signals (see also Figure 2): full /t/, strong frication /t/, weak frication /t/, closure-only, and a long consonant. These five signals constitute a /t/-Ø continuum in which the first step contains the strongest phonetic information for the presence of /t/ and the fifth step (the long consonant /n/ or /s/) no information for the presence of /t/. Because consonants tend to be longer in simple codas than in complex codas, a long /s/ or /n/ can be considered as evidence for a simple coda consisting of /s/ or /n/ with no following /t/ (Lehiste, 1970).

The first signal was similar to a full /t/ with a 25-ms closure and a 45-ms transient-frication sequence. To prevent an unnatural flat line in the signal, the closure was synthesized with 20 dB amplitude of frication (AF) and a 30-dB amplitude bypass (AB) of the parallel branch of the synthesizer. At the release of the initial burst, AB was set to zero, and AF increased to 40 dB, and decreased again to 15 dB at the end of the 65-ms signal. For this and all the other target signals, amplitude changes were log linear in dB to achieve a linear amplitude envelope. The transient-frication signal was dominated by the fifth and sixth formats (55 dB) starting at 5700 Hz (bandwidth of 500 Hz) and 7500 Hz (700 Hz), respectively. These formants fell to 5200 and 6850 Hz at 65 ms. The second, third and fourth formant stayed constant throughout the transient-frication sequence at 1434 Hz (200), 2212 Hz (500), and 3840 Hz (600), respectively, and their amplitude increased from 20 to 25 dB in order to mimic the increasing low-amplitude frication in the model [t].

The second coda signal was a 65-ms frication noise, as often found in /st/ codas. The AF started and ended at 15 dB with a 40-dB maximum at 30 ms. The settings for the second, third, and fourth formant were the same as the settings in the full [t]. The fifth and sixth formants started at 5130 Hz (bandwidth: 500 Hz) and 6750 Hz (700 Hz) and fell to 4620 and 6080 Hz at the end of the signal (*A*s = 55 dB). The weak-frication signal was derived from this signal by reducing the overall amplitude of fricative noise by a factor of 5 (=14 dB). The closure-only signal was synthesized with the same settings as for the closure of the full /t/.

The final coda signal was a coda that was not followed by any signal implying a /t/. Instead, it was created by elongating the consonant /n/ by 45 ms and /s/ by 65. These durations were based on the measurements of the coda duration in the recording made of the Dutch native speaker reading the test materials. These measurements also showed that consonants in consonant clusters are shorter than in simple codas.

It is important to note that the targets *orkes* and *charman* are existing Dutch words if followed by a /t/ (*orkest*, “orchestra” and *charmant*, “charming”), whereas the targets *moeras* “swamp” and *kanon* “canon” are Dutch words, but become non-words when a /t/ is added (i.e., *moerast* and *kanont* are not Dutch words).

#### Design and procedure

There were three independent variables: (1) the coda signal (from full production to complete deletion); (2) the context preceding the coda signal (/n/ vs. /s/); (3) the lexicality manipulation: target words were existing or non-existing words if they ended in /t/. The between-subjects variable was native language and the dependent variable was the percentage of /t/-responses in each cell of the design.

The experiment was run on a standard PC running with the NESU package. Participants were tested one at a time in a sound-attenuated booth. They wore Sennheiser headphones, sat at a comfortable reading distance from the computer screen and had a two-button response box in front of them. Instructions were given in Dutch (also to the non-native participants); these informed participants that on each trial they would hear a Dutch sentence and see two words on the computer screen, one that ended in a /t/ and one that did not. They were asked to press the right button if the sentence they heard contained a target word that ended in a /t/, and to press the left button if the target word did not end in a /t/. The 300 different stimulus sentences (six target words × five coda signals × five adverbs × two subjects) were presented only once to each participant. The presentation of the sentences was random and different for every participant.

The experiment started with four practice trials. Each trial (experimental and practice trials) began with 150 ms of blank screen. Then, the response alternative without a /t/ in the coda (e.g., “orkes”) was presented in the upper left corner of the screen whereas the other (e.g., “orkest”) was presented in the upper right corner. After another 450 ms the sentence was played. From the onset of the target word, participants had 2.5 s to press one of the buttons. After responding, the chosen alternative was moved further to the respectively (left or right) upper corner of the screen while the other alternative was removed from the screen, so that the participants could see that their answer had been registered by the computer. If a participant did not react within 2.5 s, a stopwatch was shown on the screen to remind participants to respond more rapidly. The three feedback signals – no /t/ response, /t/ response, and no response – stayed on the computer screen for 1 s before the next trial started. Participants were free to take a break after each 50th trial and could continue as soon as they were ready.

### Results

**Figure 3:**
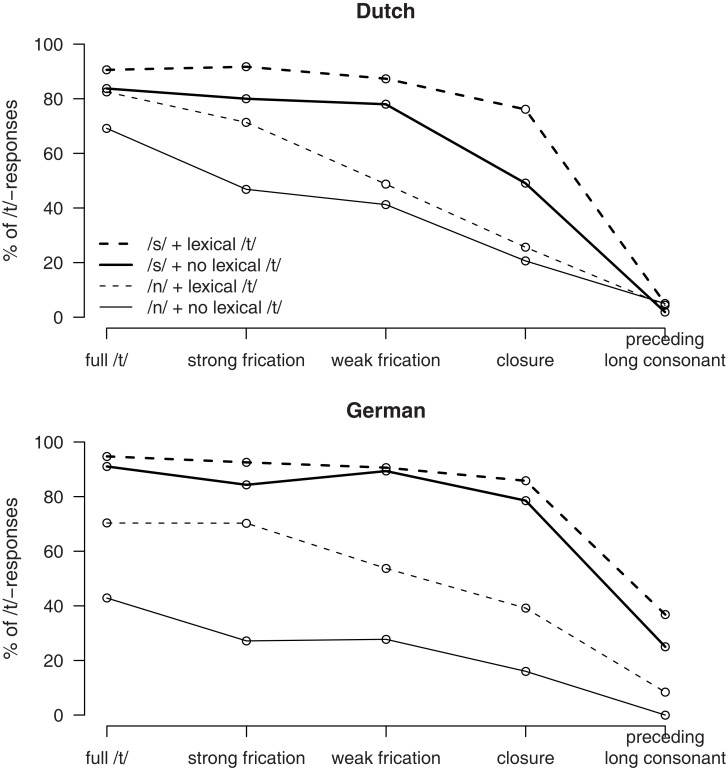
**Percentages of /t/-responses in Experiment 2 by Dutch listeners (upper panel) and German listeners (lower panel), depending on /t/-realization (*x*-axis) as well as lexical status and phonological context (different lines)**.

The results of Experiment 2 are shown in Figure 3. A comparison of the panels for native Dutch listeners and non-native German listeners shows that the overall pattern is quite similar. Participants give, independent of native language, more /t/ responses if there is more acoustic-phonetic evidence for a /t/ (an effect of the /t/-Ø continuum), if assuming a /t/ results in an existing word (an effect of Lexicality), and if the preceding context is /s/ rather than /n/.

Statistical analysis of Experiment 2 made use of an analysis of variance (ANOVA) using the logistically transformed proportion of /t/ responses (computed for each listener and each cell of the design) with Native Language, Coda Signal (from full production to complete deletion), Preceding Context (/n/ vs. /s/), and Syntax (/t/ or no /t/ predicted) as factors^1^. The results of this overall ANOVA are given in Table 2. Overall, there were main effects of Preceding Context, Lexicality, and Target; more /t/-responses were given after /s/ than after /n/, and more /t/ responses were given if an existing word resulted, and more /t/-responses for signals with more evidence for /t/. There was no main effect of native language. Overall, the effect of Preceding Context was larger for the German listeners. However, these effects were moderated by various interactions. Note, however, that the interaction of Native Language and Lexicality was not significant, that is, the German listener do not rely more strongly on lexical cues. To gain insight into the nature of the various interactions, we also examined the effects of Native Language, Preceding Context and Lexicality on all five levels of Coda Signal. Table 3 shows the resulting *F*-values and their significance level.

**Table 2:** **Analysis of variance table for Experiment 2**.

Effect	df	*F*	*p*
Native language	(1, 30)	0.76	0.3897
Target	(4, 120)	183.30	0.0000
Native language × target	(4, 120)	6.36	0.0001
Preceding context	(1, 30)	108.22	0.0000
Native language × preceding context	(1, 30)	10.38	0.0031
Lexicality	(1, 30)	28.32	0.0000
Native language × lexicality	(1, 30)	1.23	0.2769
Target × preceding context	(4, 120)	17.19	0.0000
Native language × target × preceding context	(4, 120)	1.28	0.2823
Target × lexicality	(4, 120)	5.52	0.0004
Native language × target × lexicality	(4, 120)	0.21	0.9334
Preceding context × lexicality	(1, 30)	6.06	0.0198
Native language × preceding context × lexicality	(1, 30)	7.87	0.0087
Target × preceding context × lexicality	(4, 120)	3.53	0.0093
Native language × target × preceding context × lexicality	(4, 120)	1.83	0.1275

For the full /t/, the three-way interaction between Lexicality, Native Language, and Preceding Context was analyzed by evaluating the effect of Lexicality for each combination of Preceding Context and Native Language. This shows that the effect of Lexicality was only significant for the German participants in the /n/ context [*F*(1, 15) = 11.5, *p *< 0.01], while there was a trend for Dutch participants in the same context [*F*(1, 15) = 4.3, *p *= 0.55] and no effect of Lexicality in the /s/ context [German: *F* < 1, Dutch, *F*(1, 15) = 1.4, *p* > 0.2]. For the second step, there was neither a main effect of Native Language nor an interaction, but significant effects of Preceding Context and Lexicality and their interaction (see Table 3). Figure 2 shows the source of the interaction, the effect lexicality is smaller in the /s/-than in the /n/-context. For step 3, there are only the main effects of Preceding Context and Lexicality. For Step 4, the main effect are accompanied by a three-way interaction, which was again was analyzed by evaluating the effect of Lexicality for each combination of Preceding Context and Native Language. This showed that the only the Dutch were affected by Lexicality in the /s/-Context [*F*(1, 15) = 19.1, *p *< 0.001] and only the German in the /n/-context [*F*(1, 15) = 19.1, *p *< 0.001]. For step 5, there is a main effect of Native Language with more /t/-responses by German participants an interaction of Native Language and Preceding context as only the German participants [*F*(1, 15) = 20.1, *p *< 0.001] but not the Dutch participants (*F* < 1) are influenced by the Preceding Context.

**Table 3:** ***F*-values [df = (1,30)] for the analysis of the separate steps of the /t/-Ø continuum in Experiment 2**.

Effect	Coda signal				
	Full /t/	Strong frication	Weak frication	Closure	Long consonant
Native language	2.1	0.6	0.1	7.5*	13.6*
Preceding context	32.3**	62.9**	85.5**	109.5**	19.8**
Native language × preceding context	9.4**	3.6	2.3	2.6	22.5**
Lexicality	11.8**	49.5**	8.7**	19.5**	6.6**
Native language × lexicality	0.9	1.2	0.2	0.2	2.7
Preceding context × lexicality	11.5**	10.3**	1.7	0.1	0.0
Native language × preceding context × lexicality	4.5*	1.5	2.8	10.9**	1.1

**p < 0.01; *p < 0.05.

In summary, we observed that both groups behave qualitatively similar with some quantitative differences. It is not the case that some variables influenced only one group of listeners. However, there is a tendency for German listeners to give more /t/-responses when there is little evidence for a /t/ (i.e., for the three coda signal at the “Ø” end of the /t/-Ø continuum). Moreover, there are some interactions that indicate that the Native Language influenced the degree of the effects. The effect of Preceding Context is consistently larger for German than for Dutch participants. However, there is little evidence to suggest that German participants rely overall more strongly on lexical cues to the presence of /t/. Even though there are interactions involving Lexicality and Native Language, these are not consistently in the same direction with a larger effect of Lexicality for the German listeners.

### Discussion

The results of Experiment 2 indicate that German listeners are able to make use of the phonological cues to the presence of /t/. Even though they tend to perform less categorically and, as a consequence, give more /t/-responses when there is little evidence for a /t/, they do not show a pattern of less reliance on phonological cues and more reliance on lexical cues. In fact, they even are more strongly influenced by phonological context than the Dutch listeners.

This indicates that the German listeners are generally well able to deal with /t/-reduction in their second language Dutch. This ability may be grounded in the experience German listeners have with /t/-reduction in their native language. However, there is another account for the current finding. The relatively native-like performance of German learners may simply be due to them having learned the Dutch reduction pattern rather than transfer of knowledge from their L1.

These accounts can be distinguished by looking at the pattern for verbs. If German learners are indeed able to learn the pattern of /t/-reduction in their L2 Dutch, they should also show near-native-like performance in verbs. If, however, differences between L1 and L2 influence the mastery of a casual speech process in a second language, German learners should be less proficient in dealing with /t/-reduction in verbs, where their L1 knowledge does not match the pattern of the L2 target.

## Experiment 3

### Method

#### Participants

Sixteen native speakers of German, none of whom had participated in the previous experiments, took part in Experiment 3. Participants were from the same participant pool as those of Experiment 2. To compare these data with the native pattern, control data of 21 native speakers of Dutch were taken from Tuinman et al. (submitted).

#### Materials

For this experiment, three Dutch verbs with a stem ending in /n/ and three verbs with a stem ending on /s/ were chosen with a matched lemma log frequency using CELEX (Baayen et al., 1995). These verbs were placed in sentences and a male native speaker of Dutch (the same speaker as in Mitterer and Ernestus, 2006) recorded the sentences several times. Table 4 shows the combinations of the target verbs and preceding and following words. According to the reading list, the verbs in the sentences were pronounced with or without a final /t/. These utterances were used as templates for synthesis via a Klatt synthesizer (Klatt, 1980). Stimuli were created in a similar fashion to Mitterer and Ernestus, just as in Experiment 2. Formants and bandwidths were measured at the beginning, in the middle and at the end of each segment and at major formant transitions point within segments. When the formant could not be estimated reliably by Linear Predictive Coding (using Praat, Boersma and Weenink, 2005), values recommended by Klatt (1980) were chosen. Additionally, nasalization for a nasal was carried over into the vowel before it was reduced completely, and anticipated for postvocalic nasals to match the natural utterances. Parameters for synthesis were generated by interpolating linearly between measurements points. Occasionally, values were altered in order to prevent clicks and other transients in the synthesized signal, which can occur if control parameters change too quickly. Amplitude values were iterated to imitate the amplitude envelope of the natural utterances.

**Table 4:** **Sentence frame for the stimuli in Experiment 3**.

Connection word	Subject	Target word	Adverb
Maar /ma:r/	zij /sεI	blaas /bla:s/ (1.62)	nauwelijks /’nauυeleks/
		kreun /krØ:n/ (1.46)	langzaam /’laηz:m/
		bloos /blo:s/ (1.21)‥t	moeizaam /’muzj:m
	Ik /Iκ/	zoen /zun/ (1.26) …Ø	soms /’scms/
		ren /rεn/ (1.95)	vaak /’va:k/
		kus /kus/ (1.75)	

*English translations are *Maar* “but,” *ik* “I,” *zij* “she,” *blaas* “blow,” *kreun* “moan,” *bloos* “blush,” *zoen* “kiss,” *ren* “run,” kus “kiss,” nauwelijks “hardly,” langzaam “slowly,” moeizaam “with difficulty,”, soms “sometimes,” vaak “often.” The numbers between brackets are the lemma log frequencies for the words taken from CELEX (Baayen et al., 1995)*.

The verb stems were followed by one of five different synthesized signals for the coda, just as in Experiment 2 (see also Figure 2). The long consonant target signals were adapted to the following adverb to make the transition as natural as possible between the target verb and the subsequent adverb. Thus, for the long consonant /n/ the amplitude remained high if another nasal followed (e.g., if “nauwelijks” or “moeizaam” was the following adverb), but it fell before other phonemes. For the long consonant /s/, the amplitude did not fall before another /s/ (e.g., if “soms” was the following adverb), but decreased before other phonemes. A 25-ms closure was inserted after “Maar ik” and a 50-ms closure before verbs starting with a /k/ (e.g., “kus”) to make the synthesized materials more like the natural utterances.

Thus, there were five coda signals for the target verbs: full /t/, strong frication /t/, weak frication /t/, closure-only, and a long consonant. All these different signals were put together with the six target verbs resulting in 30 stimuli for which the participants decided whether they heard a coda with or without a /t/. The 30 stimuli were placed in sentences starting with either “Maar ik …” or “Maar zij…” and five adverbs following the target words. This resulted in 300 different sentences (see also Table 3).

#### Design, procedure, and analysis

There were three within-subject independent variables for Experiment 3: (1) the coda signal (from full production to complete deletion); (2) the context preceding the coda signal (/n/ vs. /s/); (3) the syntactic manipulation: target words would be grammatically correct or not if ending in /t/. The between-subjects variable was native language and the dependent variable was the logistically transformed proportion of /t/-responses for each listener in each cell of the design. Values of one and zero were replace by the mean of one and zero and the respective next highest or lowest possible value, in line with the suggestions by Macmillan and Creelman (1991). The procedure of the experiment was as described for Experiment 2.

### Results

**Figure 4:**
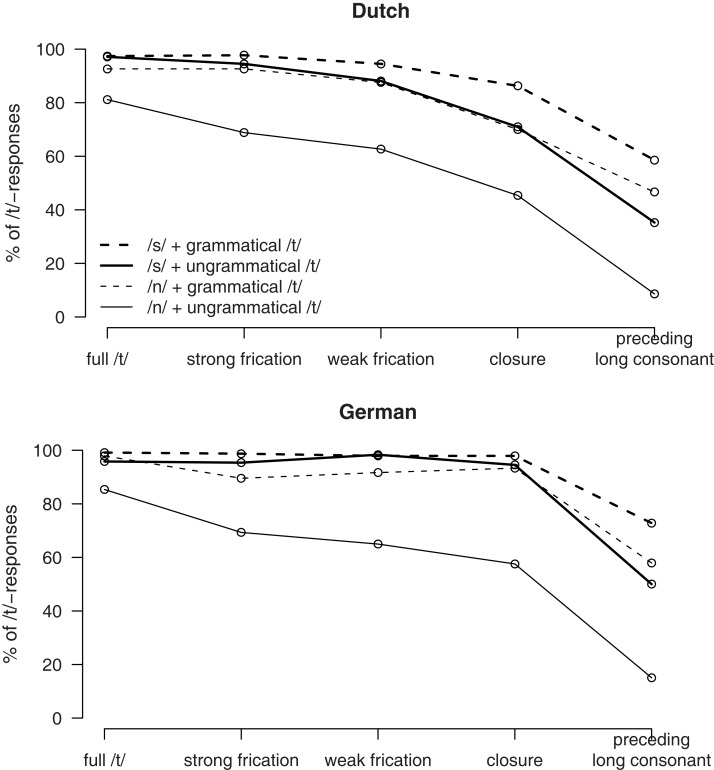
**Percentages of /t/-responses in Experiment 3 by Dutch listeners (upper panel) and German listeners (lower panel), depending on /t/-realization (*x*-axis) as well as syntactic condition and phonological context (different lines)**.

The results of Experiment 3 are presented in Figure 4. As can be seen in Figure 4, there are similar types of main effects for Dutch (left panel) and German (right panel) listeners. Listeners gave more /t/-responses with more /t/-like signal, if the syntax prescribes a /t/, and if preceding phonological context triggers /t/-reduction (/s/ > /n/). However, there are also clear differences. The German participants hardly ever give a no-/t/ response if either the syntax or the preceding phonological context creates a bias toward /t/. The identification functions stay near ceiling for the first four levels of the /t/-Ø continuum in all conditions except the one in which neither the syntax nor the phonological context bias toward a /t/-percept.

Analysis of Experiment 3 made use of an ANOVA on the logistically transformed proportions of /t/ responses over listeners for each cell of the design, the results of which are displayed in Table 5. There was an overall significant tendency for more /t/-responses by German (81.2%) than Dutch (73.9%) participants. This tendency, however, was not of the same size in all conditions, that is, it was moderated by various interactions with the other experimental variables, including a four-way interaction. To understand the nature of these interactions, we examined the effects of Native Language, Preceding Context and Syntax on all five levels of Coda Signal separately. The results of this procedure are displayed in Table 6. For all levels, there are main effect of Syntax and Preceding Context in the expected directions (more /t/ responses after /s/ and when the /t/ is syntactically prescribed) and an under additive interaction of these two factors, so that the effect Syntax was smaller in the Preceding Context condition /s/, which already leads to more /t/ responses.

**Table 5:** **Analysis of variance table for Experiment 3**.

Effect	Df	*F*	*p*
Native language	(1, 35)	6.63	0.0144
Target	(4, 140)	234.89	0.0000
Native language: target	(4, 140)	8.33	0.0000
Preceding context	(1, 35)	74.45	0.0000
Native language: preceding context	(1, 35)	0.24	0.6292
Syntax	(1, 35)	106.06	0.0000
Native language: syntax	(1, 35)	0.01	0.9086
Target: preceding context	(4, 140)	6.38	0.0001
Native language: target: preceding context	(4, 140)	0.93	0.4509
Target: syntax	(4, 140)	21.64	0.0000
Native language: target: syntax	(4, 140)	0.61	0.6567
Preceding context: syntax	(1, 35)	63.20	0.0000
Native language: preceding context: syntax	(1, 35)	1.37	0.2492
Target: preceding context: syntax	(4, 140)	2.05	0.0905
Native language: target: preceding context: syntax	(4, 140)	4.28	0.0027


**Table 6:** ***F*-values [df = (1,35)] for the analysis of the separate steps of the /t/-Ø continuum in Experiment 3**.

Effect	Coda signal				
	Full /t/	Strong frication	Weak frication	Closure	Long consonant
Native language	0.58	0.01	2.78	20.14**	6.35*
Preceding context	24.93**	49.43**	35.49**	40.94**	88.22**
Native language × preceding context	1.43	0.39	0.48	0.34	0.47
Syntax	17.69**	45.29**	54.40**	74.66**	93.14**
Native language × syntax	0.62	0.29	0.93	0.00	0.06
Preceding context × syntax	14.64**	30.05**	51.33**	26.60**	29.39**
Native language × preceding context × syntax	0.16	0.47	2.41	10.58**	0.04

**p < 0.01; *p < 0.05.

Effects of Native Language appeared at the Ø end of the /t/-Ø continuum. For both the closure-only and the long consonant target, there was a main effect of Native Language, with more /t/ responses by the German listeners (85.8 and 49.0%) than the Dutch listeners (68.3 and 37.4%). For the closure-only target there this main effect of Native Language was qualified by a three-way interaction with Preceding Context and Syntax. This interaction was caused by a difference between Dutch and German listeners for all combinations of Preceding Context and Syntax [*t*_min_(35) > 3, *p *< 0.01] except the combination of /n/ context and a first person inflection requiring no /t/ [*t*(35) = 1.4, *p *> 0.1].

### Discussion

The results of Experiment 3 show clear differences between native and non-native listeners. The non-native German listeners gave more /t/-responses than the Dutch participants. Moreover, they were less influenced by acoustic detail and more by syntactic constraints than the native Dutch listeners. Figure 4 shows that, if grammar prescribes it, German listeners became insensitive to phonetic detail information. There was hardly any influence of type of Coda signal on the amount of /t/-responses for the first four steps of the /t/-Ø continuum. In contrast, the effect of the Syntax variable tends to be larger for the non-native German than for the Dutch listeners. Put differently, German listener are less sensitive to phonological variables and rely more strongly on higher-level constraints; a result that contrasts with that of Experiment 2.

Importantly, the fact that non-native listeners behave quite differently than native listeners in Experiment 3 allows us to answer the question that motivated this experiment. Is the relatively good performance of German learners in Experiment 2 due to learning of the non-native pattern or due to transfer from the L1? If the first were the case, they should perform relatively well independent of differences between the production patterns in their L1 and their L2. However, when there are subtle differences in how /t/-reduction patterns in the two languages, German learners do not show a relatively native-like performance. This seems to indicate that the relatively good performance in Experiment 2 is due to transfer from the L1.

Transfer alone, however, cannot explain differences between Dutch and German listeners because the production pattern showed less reduction of /t/ in German than in Dutch verbs. Simple transfer should hence lead to less /t/-responses by German listeners. A speculative account of the data is that the German listeners may have learned that reductions are more likely in Dutch than in German, but failed to appreciate the exact pattern of the difference. As a consequence, they overcompensate for /t/-reduction in Dutch verbs and tend to give more /t/-responses than native listeners. Obviously, additional data is necessary to verify this *post hoc* explanation for how non-native listeners may cope with unfamiliar L2 casual speech processes.

## General Discussion

In three experiments, we investigated how L2 learners deal with a casual speech process that they are familiar with from their L1. The case under study was /t/-reduction in Dutch, and its perception by German learners of Dutch. Experiment 1 was necessary to establish to what extent /t/-reduction in Dutch and German patterns similarly, and thus, whether German learners could rely on their L1 knowledge in dealing with /t/-reduction in Dutch. If /t/ is a verbal inflection, /t/-reduction differs between the languages, with /t/-reduction being especially likely in Dutch if the /t/ is preceded by an /s/. In German, in contrast, /t/-reduction was in this case not conditioned by the preceding phonological context. If /t/ was part of the word’s stem – and hence not a verbal inflection – both languages showed similar patterns with more /t/-reduction after /s/ than after /n/.

Experiments 2 and 3 then investigated to what extent these similarities and differences are reflected in perception. Experiment 2 found that German learners behaved quite similarly to native listeners for non-morphological /t/. German listeners made use of phonological cues to the presence of /t/ and did not rely more strongly on lexical knowledge than Dutch listeners. Experiment 3 investigated the perception of /t/-reduction for morphological /t/ in verbs. In this case, there were clearer differences between native Dutch listeners and German learners of Dutch. The German learners relied more strongly on grammatical cues and paid less attention to phonological cues.

How do these results relate to the theoretical question raised in the Introduction: Does familiarity with L2 casual speech process – from the L1 – help the L2 listener? The results indicate that this is the case. In compensation for /t/-reduction in Dutch, German learners perform more native-like on nouns than on verbs, mirroring a production pattern that is more similar for nouns than for verbs.

However, there is another possibility to account for the relatively good performance of the German listeners. Maybe /t/-reduction is very easy to deal with for a German – or in fact any other – listener independent of language experience. The process of /t/-reduction occurs in several different languages and may be constrained by universal perceptual biases (Mitterer et al., 2008), so that the reduction of /t/ is more difficult to notice – and therefore less problematic for word recognition – after /s/ than after /n/.

With this line of reasoning, we join a lively debate about the role of native language learning in compensation for casual speech processes. Gow and Im (2004), for instance, argued that language experience is not necessary for listeners to compensate for assimilation, based on the finding that native and non-speakers of Hungarian and Korean dealt similarly with phonological assimilation that were peculiar to Hungarian or Korean. A similar finding has been reported by Mitterer et al. (2006b) who showed that Dutch listeners can compensate for Hungarian liquid assimilation, despite the fact that no similar process exists in Dutch. However, the data in Mitterer et al. (2006a) show a more nuanced picture. Next to language independent effects, which made the consequences of the assimilation difficult to perceive, language experience seem to help listeners with the potentially ambiguous stimuli arising from assimilation. A similar mixture of language-dependent and independent effects was reported by Darcy et al. (2007) for voicing assimilation in French. For the case of /t/-reduction, the observed differences between native Dutch listeners and German L2 learners indicate that compensation is not independent of language experience (see also Mitterer et al., 2008). This is in line with the finding that lexical effects seem to be more pronounced in compensation for /t/-reduction than in compensation for assimilation (Mitterer and Ernestus, 2006), converging on the conclusion that compensation for /t/-reduction relies heavily on higher-level processing.

As a consequence, non-native listeners show worse performance, when they cannot rely on L1 knowledge. German learners can rely on L1 knowledge for compensation for /t/-reduction in nouns and adjectives, and they seem to do so quite successfully. There were only minor difference in how Dutch and German listeners compensated for /t/-reduction in this case. However, German listeners cannot rely on L1 knowledge when dealing with the reduction of a /t/ that is a verbal inflection, where the production data revealed differences between Dutch and German. In other words, with fewer similarities between the casual speech process in the L1 and the L2, it becomes harder to effectively compensate for it. Or, vice versa, a casual speech process in the L2 is relatively easy if it occurs in a similar fashion in the L1.

This line of reasoning is buttressed if we look at a case in which a casual speech process does not exist at all in the native language. As mentioned in the Introduction, Tuinman et al. (2011) investigated the process of /r/-intrusion in British English – a process that does not occur in Dutch – and found that Dutch listeners showed a completely different pattern than native British English listeners. This study employed a 2AFC task, and listeners had to decide whether a phrase such as “saw(r)ice” meant “saw rice” or “saw ice.” Tuinman et al. varied the duration of the [r], and British English listeners showed a great sensitivity to this subphonemic detail, with a steep identification function over the /r/-duration continuum. The identification function of the Dutch learners, however, was essentially flat. The Dutch performed somewhat better if there was an orthographic cue to the presence of /r/, in a phrase such as “more ice.” (Note that in British English, “saw” and “more” rhyme, that is, “more” is canonically produced without a final /r/.) In this case, the decision was between a “singelton /r/” (“more ice”), and a geminate /r/(“more rice”) – a distinction Dutch listeners are familiar with from their native language (“meer reis” vs. “meer ijs”) – and Dutch listeners showed a more categorical identification function, that was, however, still not as steep as the identification function by British English listeners.

The data points discussed seem to converge on the conclusion that connected speech processes pose similar problems in learning a second language as new phonemes do. Processes that are unique to the L2 (e.g., /r/-insertion in British English for Dutch learners) lead to major perceptual problems. Processes that subtly differ between the L1 and the L2 lead to moderate problems (e.g., /t/-reduction in Dutch verbs for German listeners). Finally, processes that are quite similar in the L1 and L2 (.e.g., /t/-reduction in nouns in Dutch and German) are easy to master for L2 learners.

## Conflict of Interest Statement

The authors declare that the research was conducted in the absence of any commercial or financial relationships that could be construed as a potential conflict of interest.

## Footnotes

^1^This change in analysis method from mixed-effect models of an ANOVA is motivated by the more complex structure of this experiment with four factors. A mixed-effect model with a maximal random effect structure did not converge. Note that some advocates of mixed-effect models (e.g., Dixon, 2008) for proportions agree that an ANOVA with logistically transformed proportions is statistically valid. Values of 0 and 1 were replaced by the mean of 0 and 1 and the second lowest and highest possible value, respectively (c.f. Macmillan and Creelman, 1991).
